# Supplementary material for: Complex‐centric proteome profiling by SEC‐SWATH‐MS
Source: Mol Syst Biol. 2019 Jan 14;15(1):e8438. doi: 10.15252/msb.20188438 (PMC6346213; doi:10.15252/msb.20188438)
Supplement: Supplementary file 8 — Dataset EV7 [file MSB-15-e8438-s008.zip › feature_plots_string/P12814.pdf]

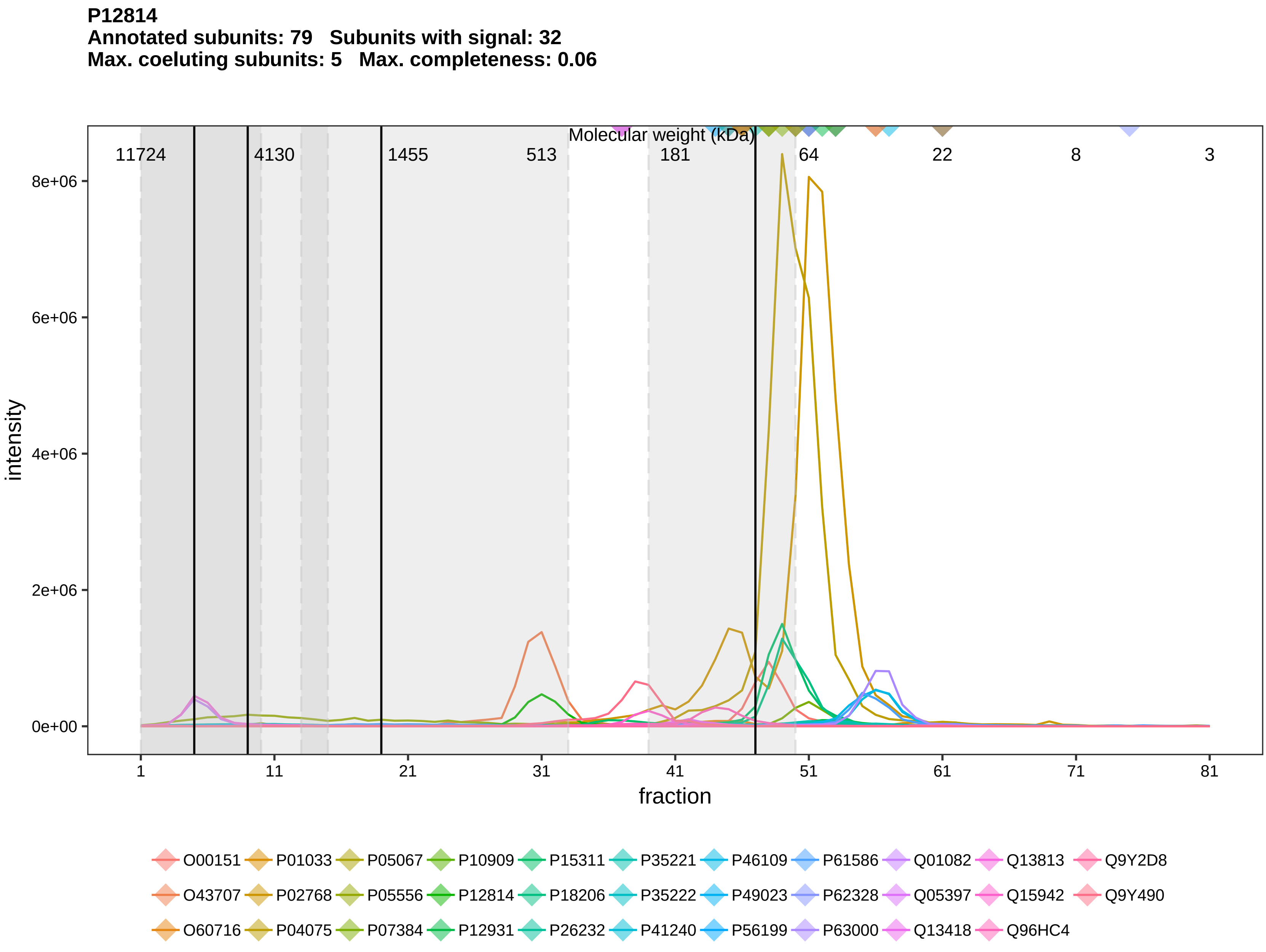

P12814
Annotated subunits: 79 Subunits with signal: 32
Max. coeluting subunits: 5 Max. completeness: 0.06
Molecular weight (kDa)
11724
4130
1455
513
181
64
22
8
3
intensity
8e+06
6e+06
4e+06
2e+06
0e+00
fraction
1
11
21
31
41
51
61
71
81
O00151
O43707
O60716
P01033
P02768
P04075
P05067
P05556
P07384
P10909
P12814
P12931
P15311
P18206
P26232
P35221
P35222
P41240
P46109
P49023
P56199
P61586
P62328
P63000
Q01082
Q05397
Q13418
Q13813
Q15942
Q96HC4
Q9Y2D8
Q9Y490
